# Evaluation of a Suicide Prevention Program Encompassing Both Student and Teacher Training Components

**DOI:** 10.1027/0227-5910/a000862

**Published:** 2022-05-12

**Authors:** Katharina Bockhoff, Wolfgang Ellermeier, Simone Bruder

**Affiliations:** ^1^ Institute of Psychology, Technical University of Darmstadt, Germany; ^2^ Darmstadt Children’s Hospital Princess Margaret, Darmstadt, Germany

**Keywords:** suicide prevention in schools, psycho-educational training, gatekeeper training, teachers, students

## Abstract

**Abstract.** *Background:* Although suicide prevention programs have been shown to change suicide-related knowledge and attitudes, relatively little is known about their effects on actual behavior. *Aims:* Therefore, the focus of the present study was on improving participating school staff’s practical and communication skills. *Method:* Suicide prevention workshops for students in grades 8–10 (*N* = 200) and a gatekeeper training program for school staff (*N* = 150) were conducted in 12 secondary schools in Germany. Schools were alternately assigned to one of three interventions (staff, students, or both trained) or to a waitlist control group. *Results:* School staff undergoing the training showed increased action-related knowledge, greater self-efficacy when counseling students in need and augmented counseling skills, and also had more conversations with students in need. Although students participating in the workshops did not seek help more frequently, they provided help to their peers more often in the conditions in which both students and school staff or only the latter had been trained. *Limitations:* The generalizability of the results is constrained by high dropout rates due to the COVID-19 pandemic and the relatively small sample size. *Conclusion:* A combination of suicide prevention programs for school staff and students appears to be most effective.

Suicide is the second main cause of death among adolescents (WHO, 2018). According to a German survey, 14.4% of 14- to 15-year-olds reported suicide-related thoughts (Brunner et al., 2007) and 8% reported a nonfatal suicide attempt (Kaess et al., 2011). Similar results have been found in the United States by Lieberman, Poland, and Cowan (2006) who estimated that in a typical high school classroom, three students had a nonfatal suicide attempt in the past year.

Schools provide the ideal setting for suicide prevention programs (King et al., 2011). Teachers may function as gatekeepers who recognize suicide warning signs, ask accurate questions, and are able to identify students at risk and refer them to appropriate care structures (Quinnett, 2012).

The best-known gatekeeper program, QPR (Question, Persuade, Refer) was developed by Quinnett (2012), and has been evaluated on several occasions (e.g., Reis & Cornell, 2008). The results show that participating school staff increased their knowledge about risk factors and warning signals (Groschwitz et al., 2017; Robinson et al., 2013; Scouller & Smith, 2002; Wyman et al., 2008), were more proactive in addressing students, and, after training was complete, and were better at recognizing those at risk (Isaac et al., 2009). Self-efficacy improved as well (Keller et al., 2009) and the confidence in their counseling skills was strengthened (Groschwitz et al., 2017; Robinson et al., 2013; Wyman et al., 2008), but not the number of interactions with students in need (Susanne Condron et al., 2015). Robinson et al. (2008), however, pointed out that little is known about the effects of a gatekeeper training on daily crisis management. A specific skills training program might facilitate more direct communication and interaction with students (Wyman et al., 2008). Therefore, some kind of transformation of the theoretical knowledge into active communication with students is needed, which might be improved by role plays, for example (Cross et al., 2011).

A well-known training program for students is Action, Care, Tell (ACT) from the Signs of Suicide program (Aseltine & DeMartino, 2004; Plöderl et al., 2010). Its results show that participating students increased their knowledge about suicide-related issues and changed their attitudes toward suicide. The program made nonfatal suicide attempts less likely, but there was no significant increase in help-seeking behavior (Aseltine & DeMartino, 2004; Aseltine et al., 2007).

During the past 5 decades, numerous suicide prevention programs have been implemented (for a review, see Fox et al., 2020).

### Goals of the Study

The intervention reported here combines two approaches to suicide prevention: (1) a gatekeeper training for teachers and school social workers and (2) a psycho-educational program for students. Furthermore, (3) existing regional care structures for crisis counseling were integrated into the program. Here, we cooperated with ANNA (Alles, Nur Nicht Aufgeben – *Just do not give up*; https://projektanna.org), which is an open counseling center specializing in suicide prevention at the Darmstadt Children’s Hospital Princess Margaret.

Combining these three components is probably the most novel aspect of the present research and was emphasized to overcome weaknesses in previous interventions.

The hypotheses are as follows:

(1) The gatekeeper training for school staff improves action related knowledge, perceived self-efficacy, and counseling skills.
(2) The combination of a school staff-directed gatekeeper training and a student-directed psycho-educational suicide prevention workshop will be more beneficial than either component alone, (a) by enhancing the counselors’ competencies and (b) by inducing a greater number of supportive interactions with young people at risk of suicide.

## Methodology

### Study Design

**Figure 1:**
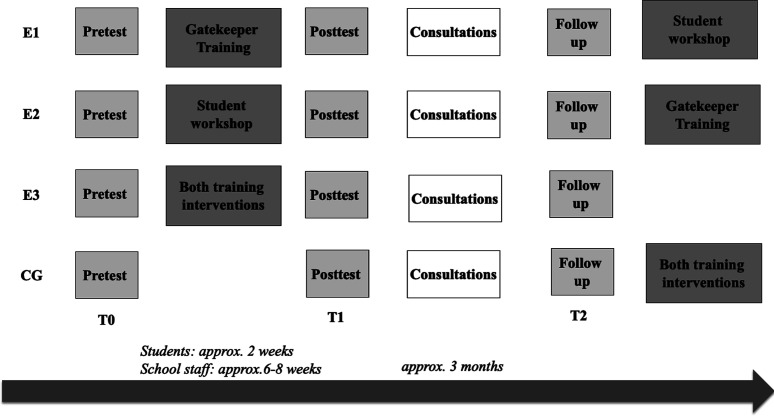
Study design and timeline.

A two-factor experimental design with the factors “student workshop” (yes/no) and “training for school staff” (yes/ no) was chosen (see Figure 1). This resulted in four study conditions: (E1) gatekeeper training for school staff, (E2) workshop for students, (E3) training programs for both, and waitlist control group (CG).

All training programs were free of charge, and the participants had to actively and voluntarily enroll. After the follow-up survey (T2), the experimental groups that had only received one type of intervention (E1 or E2) received the other type of training, and the waitlist CG received both. On three occasions (Figure 1), data were collected using paper–pencil questionnaires in all groups for students and school staff (regardless of whether training was being carried out): T0 (pretest), T1 (posttest), and T2 (follow-up test, approximately 3 months after completion of the training). The respective intervention was carried out between T0 and T1. Counseling opportunities were monitored at both T1 and T2.

### Participants

The sample was recruited from schools in the state of Hesse, Germany. Twelve schools with a total of *N* = 150 teachers and school social workers and *N* = 200 students participated from April 2019 until September 2020. The schools were assigned to the three experimental groups (E1–E3, Figure 1) or the CG in a fixed sequence by the order in which they agreed to participate in the study, with approximately three schools ending up in each of the four conditions (Table 1). The first school that agreed to participate was assigned to the first experimental condition (E1), the second school got into E2, the third school into E3, and the fourth school contributed to the waitlist CG, and so on. The study design and protocol was approved by the Central Ethics Commission of TU Darmstadt before implementation (Application No. EK 52 – 2018).

**Table 1:** Sample characteristics in the four study conditions

Variable	Group	E1	E2	E3	CG
Schools participating	All schools	3	3	4	2
*N*	School staff Students	45 31	43 67	26 52	36 50
*N* complete data sets	School staff Students	18 5	28 38	18 28	29 28
*N* gender	School staff – female School staff – male Students – female Students – male	38 7 28 3	18 8 47 18	30 12 43 9	30 6 38 12

The majority of participants in the gatekeeper training were teachers (86%, *N* = 129) and school social workers (12%, *N* = 18; Table 1). School psychologists were not recruited because they work remotely and are typically responsible for a large number of schools. Since no significant differences between these occupational groups in the variables studied surfaced at T0, their data were combined into a single group labeled “school staff.” Most of the participating school staff (77%, *N* = 116) and students (79%, *N* = 156) were female (Table 1). All student participants were at the secondary level of schooling (which is segregated in different types of schools in Germany).

Students were screened for suicidality before the intervention. This was necessary to exclude students at risk from the program and to refer them to ANNA.

To record the suicidal thoughts and behaviors, we used the first item of the Suicidal Behaviors Questionnaire – Revised (Osman et al., 2001). The criterion used was the first screening item “Have you ever thought about or attempted to kill yourself?”. Those who answered “never” or “I only had fleeting thoughts” were classified as not at risk (Forkmann et al., 2016), and all the others were classified as being at risk of suicide.

Those who were classified as at risk were required to report to a counseling center at the Childrens’ Hospital (ANNA) before participating in the workshop. There, clinicians (psychologists and psychotherapists) determined whether there was an acute risk or whether it was safe to participate. If necessary, further counseling was offered.

This conditional admission affected 23% (*N* = 46) of all participants.

### Dropouts

Because of the coronavirus pandemic, we had to stop data collection in three schools in March 2020. It was possible to resume the study in the fall of 2020 and collect more school staff training data but not to collect all of the students’ data.

Of the 150 school staff, all completed the first survey, 117 the second, and 110 the third. There were 93 complete data sets, meaning that all questionnaires had been filled out.

Of the 200 participating students, 196 completed the first questionnaire, 153 the second, and 106 the third, which resulted in 99 complete data sets.

### Training Programs

With the exception of the waitlist control group, all participating schools were offered gatekeeper training for school staff or a student workshop or both during the study period. The training materials were derived from recent scientific publications. The gatekeeper training was based on Quinnett’s (2012) QPR program, supplemented with interviewing techniques and an action plan for the school staff to deal with suicidal students. In addition, further information on mental disorders (e.g., eating disorders) was provided. Staff were taught skills in recognizing students at risk at an early stage, establishing a helpful relationship, and asking students whether they had suicidal thoughts. Each 12-hour training program was conducted by two or more instructors, at least one of whom had a master’s degree in psychology, and clinical experience in working with adolescents presenting with suicidality. On average, one training program included 13–14 participants (min = 7, max = 21).

The student workshop consisted of one 4-hour training session and included the ACT principles (Aseltine & DeMartino, 2004; Plöderl et al., 2010). The psycho-educational intervention for students focused on knowledge about suicidality and psychological disorders, development of coping strategies, and the identification of warning signs and learning how to deal with them. Using film material and role plays, students actively practiced the ACT principles.

### Dependent Measures

#### Overall Acceptance of the Program

To assess the acceptance of all training programs, we used an open question “How did you like the training program?” with a 6-point Likert scale, 1 *being the best* and 6 *being the worst rating* (German school grades).

#### Students

For measuring the students’ help-seeking behavior (T1 and T2), a multiple-choice question was formulated, asking whether the student had talked to a teacher or friend when feeling *down* during the past 3 months. A similar question addressed help-giving behavior, asking whether the students had talked to a peer they were worried about in the same period. Other dependent variables (e.g., symptoms of depression) have been analyzed in a companion paper (Bockhoff et al., in press) and are not reported in the present analysis focusing on behavioral outcomes.

#### School Staff: Action-Related Knowledge

**Figure 2:**
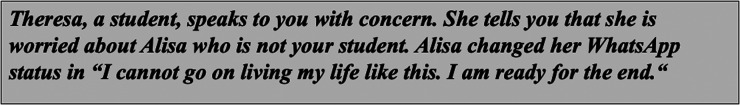
Fictional crisis situation. The vignette employed to assess action-related knowledge (at T0, T1, and T2).

For assessing action-related knowledge, a vignette representing a fictional crisis situation involving a student called Theresa (Figure 2) accompanied by a 12-item questionnaire (both developed in our clinic; Hirsch, 2019) assessed how school staff could apply action-related knowledge. Inter-rater reliability ranged between kappa = .886 and kappa = .946.

The 12 questions posed included what to say to Theresa; how to prepare, start, organize, and end the consultation; and how to find out about suicidality.

#### School Staff: Assessing Counseling and QPR Skills

A questionnaire to assess the school staff’s self-efficacy in counseling students in need was constructed by our colleagues at the University of Heidelberg. It consists of nine items (e.g., “I am confident of spotting signs of suicidality in students.”) with an internal consistency of .887.

To assess the school staff members’ self-assessed counseling skills, we developed our own questionnaire, consisting of 11 items (e.g. “I ask the student to describe the problem, without initially evaluating it.”) which had an internal consistency of .825. To measure the QPR skills (Quinnett, 2012), we developed a questionnaire with nine items (e.g. “If a student is obviously not doing well, I ask what’s going on.”) with an internal consistency of .752. For measuring the number of suicide-related interactions, a multiple-choice question asked whether the school staff member had talked to a student in need during the past 3 months.

#### Data Analysis

The statistical evaluation of the data was carried out with the statistics software SPSS 25 (IBM). Participants who – for a given measure – did not answer a question or ticked two response alternatives were excluded from further analysis. Since relatively few comparisons were made based on a priori hypotheses, an uncorrected α = 5% was chosen as the critical significance level. Where appropriate, *χ*^*2*^ tests and ANOVAs were carried out, after checking that the prerequisites had been met.

## Results

### Overall Acceptance of the Training Programs

Overall, participants were satisfied with the training programs. Students rated the training with 1.92 (*SD* = 1.028), corresponding to a B grade. Of those students who rated this item, 76.4% (*N* = 55) indicated they would definitively recommend the training and 23.6% (*N* = 17) said they might recommend it. School staff rated the training with 1.45 (first training; *SD* = .651), 1.72 (second training; *SD* = .832), and 1.56 (third training; *SD* = .915).

### Effects of the Gatekeeper Training on School Staff

**Figure 3:**
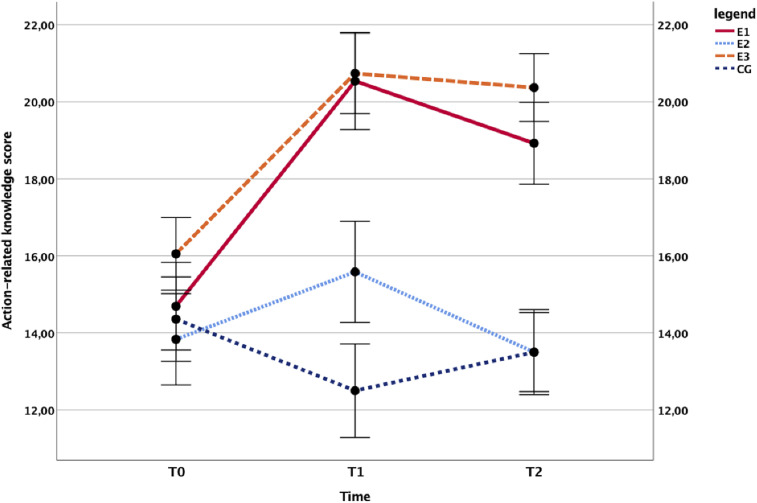
Effect of the study conditions on action-related knowledge. Time course of the school staff’s mean action-related knowledge in the trained groups (E1: *N* = 13, E3: *N* = 19, solid line) compared with untrained controls (E2: *N* = 12, CG: *N* = 14, dashed line), plotted with SEs. CG = control group.

To determine whether the gatekeeper training improved proactive behavior, a 3 × 4 mixed ANOVA was conducted with the three measuring occasions (T0, T1, and T2) constituting the repeated measure and the intervention conditions (E1, E2, E3, and CG) constituting the group factor. The main effect of time, *F*(2,108) = 10.551, *p <* .001, η^2^ = .163; the main effect of the group, *F*(3,54) = 10.386, *p* < .001, η^2^ = .366; and the group × time interaction, *F*(6,108) = 5.384, *p* < .001, η^2^ = .230, were all highly significant. In Figure 3, it is evident that action-related knowledge rapidly grows in the treated groups only and that it remains at a stable level at follow-up.

**Figure 4:**
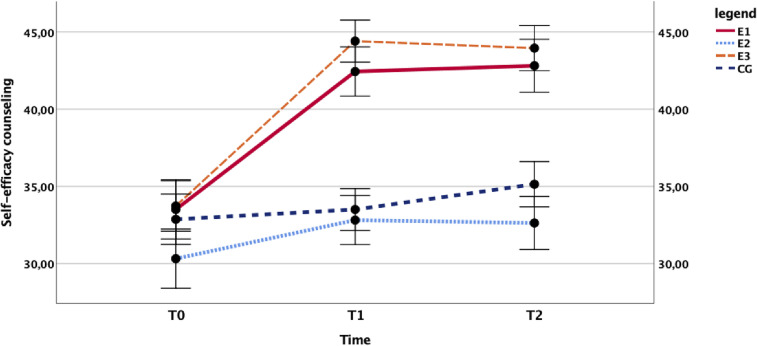
Influence of the training programs on self-efficacy. Time course of the school staff’s mean self-efficacy in counseling students in need in the trained groups (E1: *N* = 16, E3: *N* = 22) versus untrained controls (E2: *N* = 16, CG: *N* = 22). CG = control group.

School staff who completed the training developed greater self-efficacy in counseling students in need. The main effect of time, *F*(2,144) = 54.375, *p* < .001, η^2^ = .430; the main effect of the group, *F*(3,72) = 8.292, *p* < .001, η^2^ = .271; and the group × time interaction, F(6,144) = 9.194, *p* < .001, η^2^ = .277 were all highly significant, which is due to the marked increase in self-efficacy observed in the treated groups that is absent in the CGs as seen in Figure 4.

**Figure 5:**
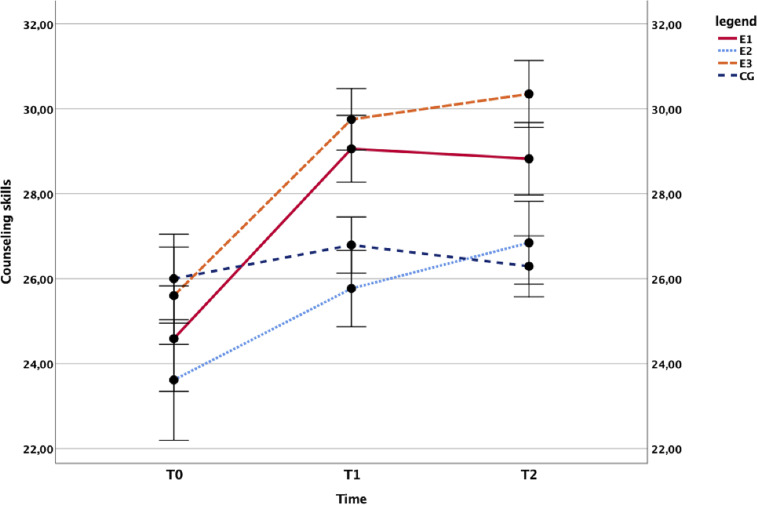
Effect of the training programs on counseling skills. Time course of the school staff’s mean counseling scores in the trained groups (E1: *N* = 17, E3: *N* = 20) compared with the untrained controls (E2: *N* = 13, CG: *N* = 24).

School staff completing the gatekeeper training also developed better counseling skills. The main effect of time, *F*(2,140) = 25.133, *p* < .001, η^2^ = .264 that of the group, *F*(3,70) = 2.978, *p* = .037, η^2^ = .113 and the interaction, F(6,140) = 3.124, *p* = .007, η^2^ = .118, were all statistically significant. This pattern of outcomes is consistent with the means plotted in Figure 5, where counseling skills increase with time in all conditions but do so to a greater extent in the intervention groups.

**Figure 6:**
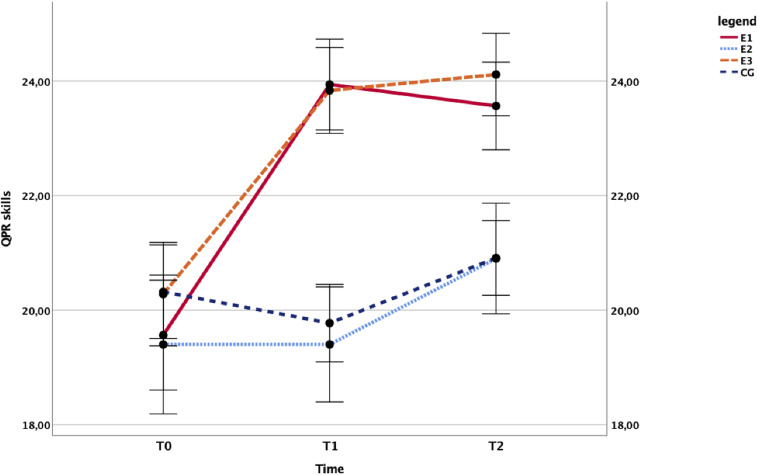
Influence of the training conditions on self-reported QPR skills. Time course of the school staff’s mean QPR (Question, Persuade, Refer) scores in the trained groups (E1: *N* = 16, E3: *N* = 18) compared with the untrained controls (E2: *N* = 10, CG: *N* = 22). CG = control group.

Furthermore, school staff participating in the gatekeeper training developed better QPR skills. The main effect of time, *F*(1.624, 100.685) = 19.417, *p* < .001, η^2^ = .238 that of the group, *F*(3,62) = 3.987, *p* = .012, η^2^ = .162 and the interaction, F(4.872, 100.685) = 5.393, *p* < .001, η^2^ = .207, were statistically significant. That is evident in Figure 6, where the self-reported QPR skills rapidly grow in the treated groups and remain at a stable level at follow-up (Figure 6).

### Interactions Between Student and Teacher Training Programs

To determine whether the implementation of a school staff training program and a student workshop at the same school do in fact interact, we checked whether the combination of teacher and student training (E3) increased problem-related interactions when compared with the single training programs (E1 and E2) or no training all (CG).

Immediately after completion of the training (T1), the number of problem-related contacts reported by the school staff did not differ significantly between intervention conditions (E1 to E3), χ^2^(6) = 6.561, *p* = .363. At the 3-month follow-up (T2), however, the (trained) school staff in groups E1 and E3 reported significantly more student contacts than staff assigned to E2, where only the students had been trained, or the untrained CG, χ^2^(6) = 29.496, *p* < .001 (Table 2).

**Table 2:** School staff’s reported problem-related interactions with students (at T2)

Trial condition	Interactions (%)	No interactions (%)	Missings (%)
E1	11 (39.3)	14 (50.0)	3 (10.7)
E2	0 (0.0)	9 (40.9)	13 (59.1)
E3	11 (40.8)	12 (44.4)	4 (14.8)
CG	2 (10.5)	14 (73.7)	3 (15.8)

When students reported their help-giving interactions (at T2, see Table 3), significantly more contacts were reported in the conditions in which both groups (E3) or only school staff (E1) had been trained; χ^2^(6) = 14.759, *p* = .022 (see Table 3). Where only students had been trained (E2), there was no difference compared to the CG.

**Table 3:** Students’ reports of interactions with peers in need

Trial condition	Conversations (%)	No conversations (%)	Missings (%)
E1	3 (60.0)	2 (40.0)	0 (0.0)
E2	12 (30.8)	19 (48.7)	8 (20.5)
E3	18 (60.0)	12 (40.0)	0 (0.0)
CG	10 (32.3)	19 (61.3)	2 (6.4)

The intervention condition did not affect how often students asked a peer for help, χ^2^(6) = 9.488, *p* = .148, or turned to school staff for help, χ^2^(6) = 3.408, *p* = .756.

## Discussion

The goal of this study was to improve suicide prevention in schools by developing, administering, and evaluating both a 12-hour gatekeeper training program for teachers and school social workers (*N* = 150) and a 4-hour workshop for students grade 8–10 (*N* = 200). These interventions had significant effects on students and school staff both at a cognitive level (e.g., self-efficacy with respect to suicide prevention) and a behavioral level (relevant counseling interactions initiated). The crucial end point criterion of reducing *suicidality* clearly falls outside the scope of this research, given the relatively small sample. Furthermore, combining both interventions in any one school revealed some interesting interactions, particularly by boosting help-giving behavior of staff and peers.

Participants in the gatekeeper training improved significantly in their action-related knowledge, their self-efficacy in counseling students in need, and their QPR skills – regardless of whether students had also been trained or not – in comparison with the waitlist CG. This is consistent with Keller et al. (2009) who postulated increased self-efficacy after QPR training. Participants in all subgroups reported that their counseling skills improved over time but to a greater extent in the groups receiving the gatekeeper training.

As to interaction effects between the two types of training, we found that school staff participating in our training (E1 and E3) initiated a significantly greater number of conversations with students in need than staff not participating in the training. Furthermore, students’ help-giving behavior increased after both groups or only school staff had been trained. In line with Aseltine et al. (2007), however, we did not find a difference in students’ help-seeking behavior as a function of training.

Because not every student in need can be identified by a peer or by school staff, and as those with a history of nonfatal suicide attempts may not believe that an adult at school can help them at all (Wyman et al., 2008), training gatekeepers may not be sufficient but will need to be supplemented by suicide prevention programs aimed at students. By analyzing the outcomes of our own student workshops, we demonstrated that particularly vulnerable students (at risk for suicidality or major depression) benefitted from the intervention. Their depressive symptoms decreased significantly (Bockhoff et al., in press).

An obvious limitation of the present study is the predominantly female sample, which prevents conclusions being drawn regarding gender differences in responsiveness to the interventions. The results may have been further biased by the self-selection of participants. Very likely, we have reached motivated young students who volunteer to talk about suicide-related issues in a group setting. Offering individual counseling may have yielded a different sample – probably at the cost of outreach. By contrast, requiring compulsory participation on a system-wide scale will be nearly impossible to implement in the near future.

Finally, practical reasons (e.g., students moving to other parts of Germany or changing schools) and most notably the COVID-19 pandemic of 2020 meant that we had considerable attrition, particularly in the student sample. While the relatively short follow-up interval (3 months) is a shortcoming of the present study, extending it further might have resulted in an even greater loss of participants at T2 due to these difficulties. Note, however, that a great number of studies with medium-to-large sample sizes have found only small effects of suicide prevention programs so far (Fox et al., 2020). This is why more innovative combinations of interventions targeting both teachers and students are needed.

This exploratory study is, to our knowledge, the first to enable comparisons between workshops for students and school staff relative to a CG. Both training interventions appeared to be effective in themselves and – in addition – to interact in fostering more interactions with students in need. This is why a combination of both kinds of training would seem to be desirable. Gatekeepers are able to identify students at risk and refer them to appropriate care structures. Those students who are not identified by trained gatekeepers among school staff may benefit from specifically tailored student workshops, thereby providing synergy in helping students to manage their crises.
